# Supplementary material for: Estimation of Parental Effects Using Polygenic Scores
Source: Behav Genet. 2021 Jan 2;51(3):264–78. doi: 10.1007/s10519-020-10032-w (PMC8093180; doi:10.1007/s10519-020-10032-w)
Supplement: Supplementary file 1 — Electronic supplementary material 1 (PDF 687 kb) [file 10519_2020_10032_MOESM1_ESM.pdf]

Estimation of parental effects using polygenic scores: Supplementary Material

Jared V. Balbona[1], Yongkang Kim[1,*], Matthew C. Keller[1,2,*]

1 Institute for Behavioral Genetics, University of Colorado Boulder

2 Department of Psychology & Neuroscience, University of Colorado Boulder

***jared.v.balbona@gmail.com *matthew.c.keller@gmail.com**

1 Path Diagrams

1.1 No Assortative Mating, Equilibrium (Observed PRS Only)

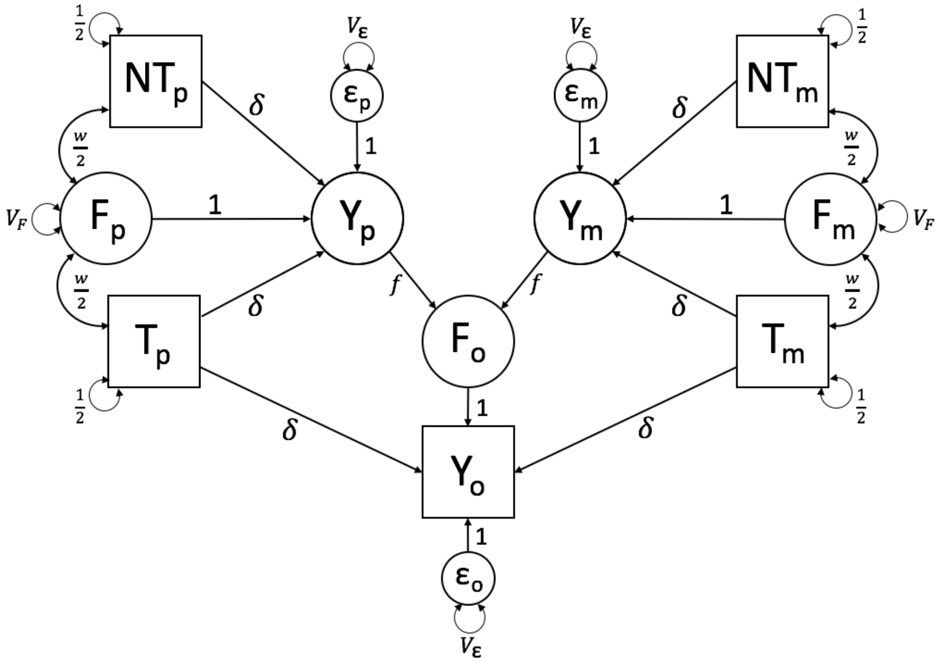

Supp. Figure 1. Path diagram modeling the effects of VT at equilibrium, assuming that the PGS explains the full trait heritability and that there is no AM.

1.2 No Assortative Mating, Disequilibrium (Observed PRS Only)

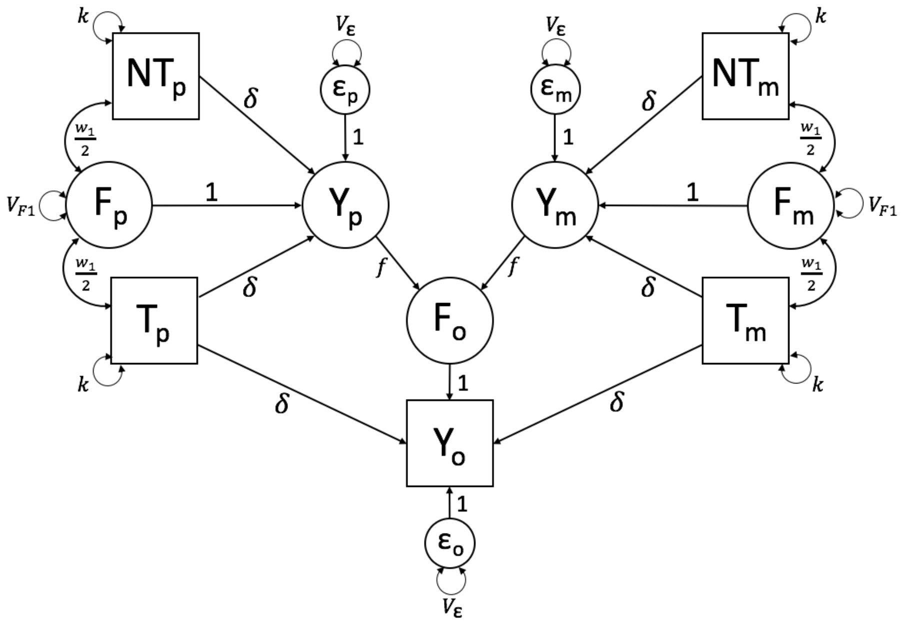

Supp. Figure 2. Path diagram modeling the effects of VT at disequilibrium, assuming that the PGS explains the full trait heritability and that there is no AM. Because the model is at disequilibrium, subscripts 1 (shown) and 2 (implied) are necessary to differentiate constraints in the parental generation from constraints in the offspring generation.

1.3 Assortative Mating, Equilibrium (Observed PRS Only)

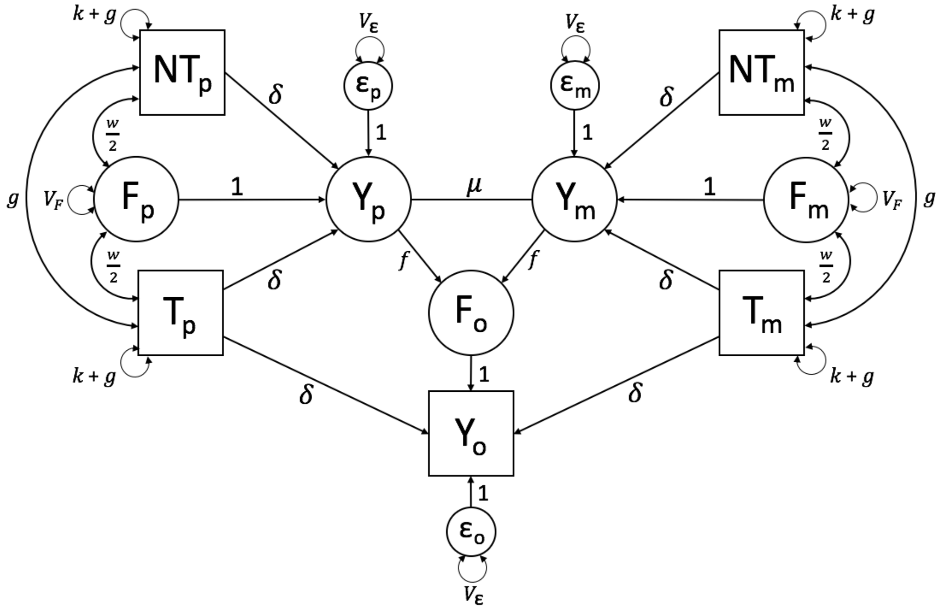

Supp. Figure 3. Path diagram modeling the effects of VT and AM at equilibrium, assuming that the PGS explains the full trait heritability.

1.4 Assortative Mating, Disequilibrium (Observed PRS Only)

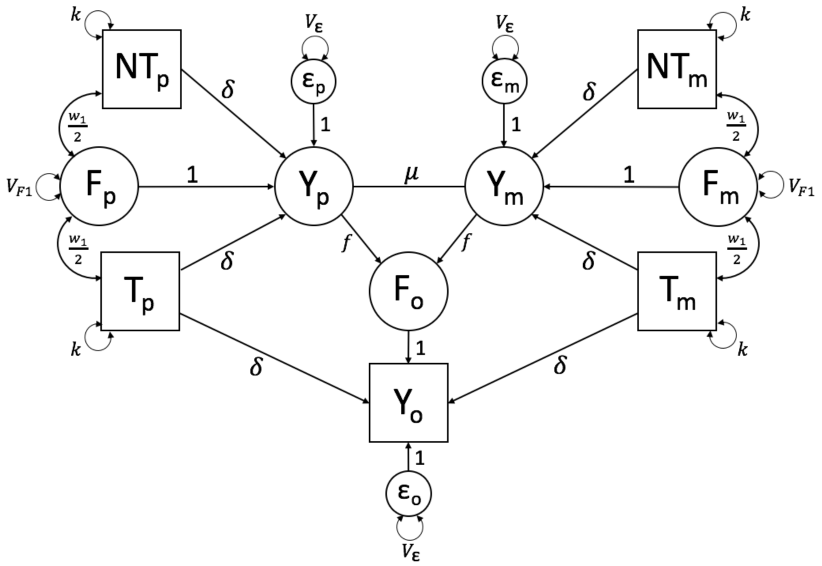

Supp. Figure 4. Path diagram modeling the effects of VT and AM at disequilibrium (after one generation of AM), assuming that the PGS explains the full trait heritability. Because the model is at disequilibrium, subscripts 1 (shown) and 2 (implied) are necessary to differentiate constraints in the parental generation from constraints in the offspring generation.

1.5 No Assortative Mating, Equilibrium (Latent and Observed PRS)

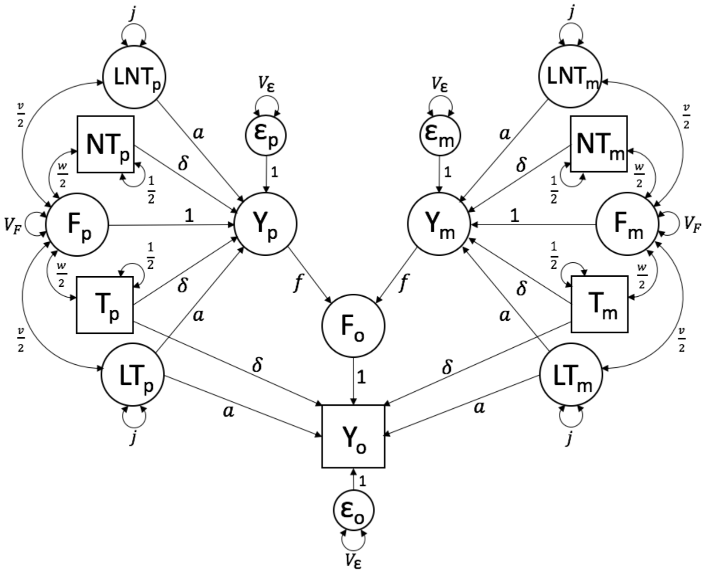

Supp. Figure 5. Path diagram modeling the effects of VT at equilibrium, assuming that there is no AM. Because Y_p and Y_m are unobserved in this Model (hence the circles used to represent them), the value of a must be assumed prior to running the model; otherwise, the model will be under-identified. Alternatively, one could use observed parental phenotypes, thereby allowing them to estimate the value of a.

1.6 No Assortative Mating, Disequilibrium (Latent and Observed PRS)

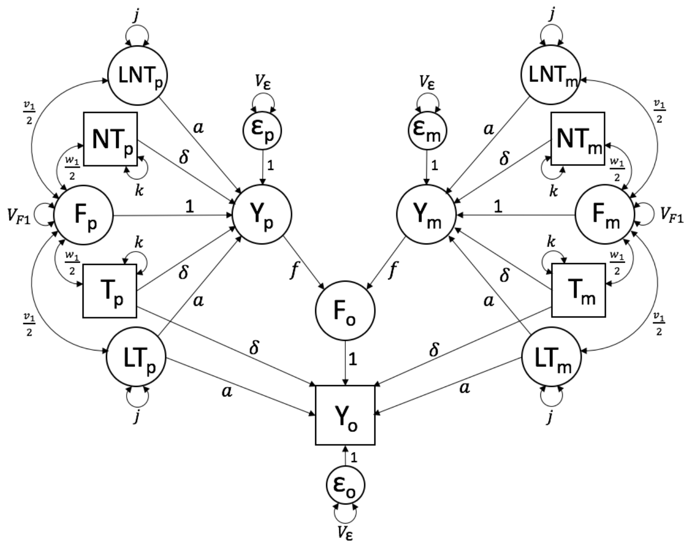

Supp. Figure 6. Path diagram modeling the effects of VT at disequilibrium, assuming no AM. Because Y_p and Y_m are unobserved in this Model (hence the circles used to represent them), the value of a must be assumed prior to running the model; otherwise, the model will be under-identified. Alternatively, one could use observed parental phenotypes, thereby allowing them to estimate the value of a. Also, because the model is at disequilibrium, subscripts 1 (shown) and 2 (implied) are necessary to differentiate constraints in the parental generation from constraints in the offspring generation.

1.7 Assortative Mating, Equilibrium (Latent and Observed PRS)

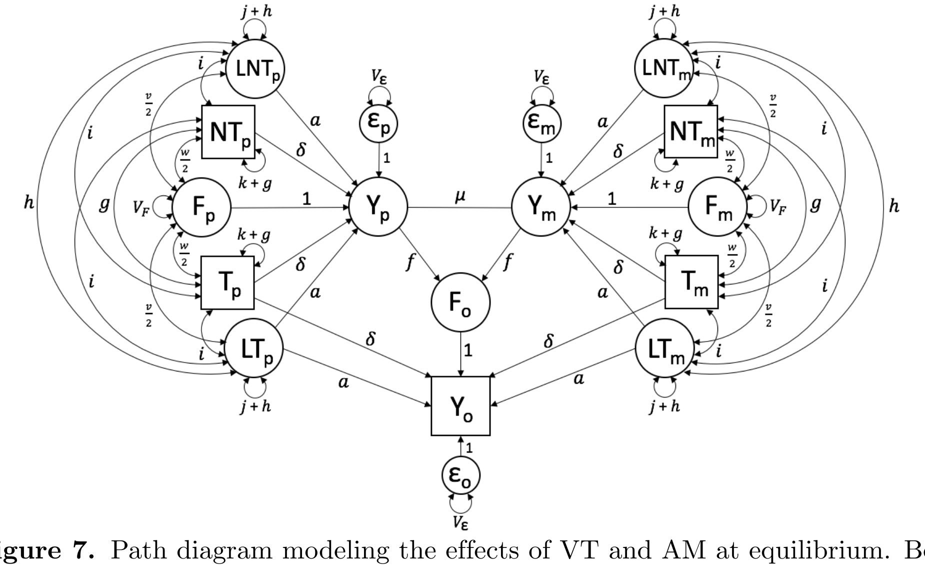

Supp. Figure 7. Path diagram modeling the effects of VT and AM at equilibrium. Because Y_p and Y_m are unobserved in this Model (hence the circles used to represent them), the value of a must be assumed prior to running the model; otherwise, the model will be under-identified. Alternatively, one could use observed parental phenotypes, thereby allowing them to estimate the value of a.

1.8 Assortative Mating, Disequilibrium (Latent and Observed PRS)

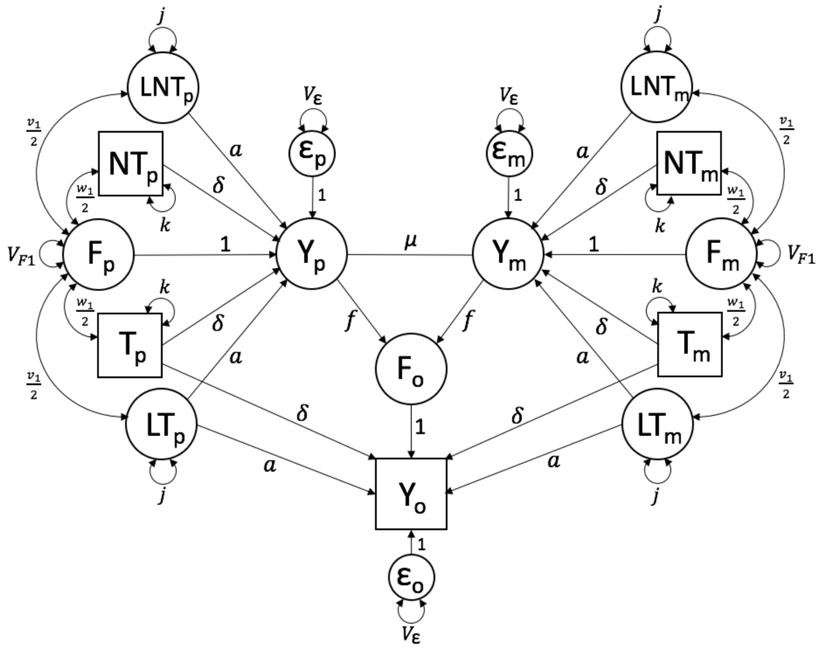

Supp. Figure 8. Path diagram modeling the effects of VT and AM at disequilibrium (after one generation of AM). Because Y_p and Y_m are unobserved in this Model (hence the circles used to represent them), the value of a must be assumed prior to running the model; otherwise, the model will be under-identified. Alternatively, one could use observed parental phenotypes, thereby allowing them to estimate the value of a. Also, because the model is at disequilibrium, subscripts 1 (shown) and 2 (implied) are necessary to differentiate constraints in the parental generation from constraints in the offspring generation.

Supp. Table I: Equilibrium Model Constraints

	**No AM, No Latent PGS's**	**No AM, Latent PGS's**	**AM, No Latent PGS's**	**AM, Latent PGS's**
Ω	$\frac{1}{2}(\delta + w)$	$\frac{1}{2}(\delta + w)$	$\delta k + \frac{1}{2}w + 2\delta g$	$\delta k + \frac{1}{2}w + 2\delta g + 2ai$
g	0	0	$\Omega^2\mu$	$\Omega^2\mu$
V_F	$2f^2V_Y$	$2f^2V_Y$	$2f^2V_Y(1 + V_Y\mu)$	$2f^2V_Y(1 + V_Y\mu)$
w	$2f\Omega$	$2f\Omega$	$2f\Omega(1 + V_Y\mu)$	$2f\Omega(1 + V_Y\mu)$
θ_{NT}	$2f\Omega$ =w	$2f\Omega$ = w	$4\delta g + 2w$	$4\delta g + 4ai + 2w$
θ_T	$\delta + 2f\Omega$ $= \delta + \theta_{NT}$	$\delta + 2f\Omega$ $= \delta + \theta_{NT}$	$2\delta k + 4\delta g + 2w$ $= 2\delta k + \theta_{NT}$	$2\delta k + 4\delta g + 4ai + 2w$ $= 2\delta k + \theta_{NT}$
V_Y	$2\delta\Omega + \delta w + V_F + V_\epsilon$	$2a\Gamma + 2\delta\Omega + av$ $+ \delta w + V_F + V_\epsilon$	$2\delta\Omega + \delta w + V_F + V_\epsilon$	$2a\Gamma + 2\delta\Omega + av$ $+ \delta w + V_F + V_\epsilon$
Γ	–	$aj + \frac{1}{2}v$	–	$aj + \frac{1}{2}v + 2ah + 2\delta i$
v	–	$2f\Gamma$	–	$2f\Gamma(1 + V_Y\mu)$
θ_{LNT}	–	$2f\Gamma$ = v	–	$4ah + 4\delta i + 2v$
θ_{LT}	–	$2aj + v$ = 2aj + θ_{LNT}	–	$2aj + 4ah + 4\delta i + 2v$ = 2aj + θ_{LNT}
h	–	0	–	$\Gamma^2\mu$
i	–	0	–	$\Omega\mu\Gamma$ $= \sqrt{g_2h_2}$
cov(Y_p, $L[N]T_m$) cov(Y_m, $L[N]T_p$)	–	0	–	$V_Y\mu\Gamma$
cov(Y_p, $[N]T_m$) cov(Y_m, $[N]T_p$)	0	0	$V_Y\mu\Omega$	$V_Y\mu\Omega$
cov(Y_p, F_m) cov(Y_m, F_p)	0	0	$V_Y\mu(\delta w + V_F)$	$V_Y\mu(\delta w + av + V_F)$
cov(F_p, F$_m$)	0	0	$\mu(\delta w + V_F)^2$	$\mu(\delta w + av + V_F)^2$
cov(Y_p, Y_m)	0	0	$V_Y^2\mu$	$V_Y^2\mu$
cov(Y_o, Y_*)	$\delta\Omega + fV_Y$	$\delta\Omega + a\Gamma + fV_Y$	$(\delta\Omega + fV_Y)(1 + V_Y\mu)$	$(\delta\Omega + a\Gamma + fV_Y)(1 + V_Y\mu)$
cov(Y_*, F_o)	fV_Y	fV_Y	$fV_Y(1 + V_Y\mu)$	$fV_Y(1 + V_Y\mu)$
cov(Y_*, F_*)	$\delta w + V_F$	$\delta w + av + V_F$	$\delta w + V_F$	$\delta w + av + V_F$

Parameter constraints under equilibrium. Here, we use the $*$ subscript to denote either p or m but not both within a single term. For example, $cov(Y_*, [N]T_*)$ can be written in the place of $cov(Y_p, T_p)$, $cov(Y_p, NT_p)$, $cov(Y_m, T_m)$, or $cov(Y_m, NT_m)$. However, $cov(Y_*, T_*)$ does not equal $cov(Y_p, T_m)$ or $cov(Y_m, T_p)$, which mix m and p in the same term.

Supp. Table II: Disequilibrium Model Constraints

	**No AM, No Latent PGS's**	**No AM, Latent PGS's**	**AM, No Latent PGS's**	**AM, Latent PGS's**
Ω_1	$\frac{1}{2}(\delta + w_1)$	$\frac{1}{2}(\delta + w_1)$	$\delta k + \frac{1}{2}w_1$	$\delta k + \frac{1}{2}w_1$
Ω_2	$\frac{1}{2}(\delta + w_2)$	$\frac{1}{2}(\delta + w_2)$	$\delta k + \frac{1}{2}w_2 + \delta g$	$\delta k + 2ai_2 + \frac{1}{2}w_2 + \delta g_2$
g_1	0	0	0	0
g_2	0	0	$\Omega_1^2\mu$	$\Omega_1^2\mu$
V_{F1}	$2f^2V_{Y1}$	$2f^2V_{Y1}$	$2f^2V_{Y1}$	$2f^2V_{Y1}$
V_{F2}	$2f^2V_{Y2}$	$2f^2V_{Y2}$	$2f^2V_{Y1}(1+V_{Y1})$	$2f^2V_{Y1}(1+V_{Y1})$
w_1	$2f\Omega_1$	$2f\Omega_1$	$2\Omega_1\mu(V_{F1} + \delta w_1)$	$2\Omega_1\mu(V_{F1} + \delta w_1 + av_1)$
w_2	$2f\Omega_2$	$2f\Omega_2$	$2f\Omega_1(1 + V_{Y1}\mu)$	$2f\Omega_1(1 + V_{Y1}\mu)$
θ_{NT}	$2f\Omega_1$ $= \mathrm{w}_1$	$2f\Omega_1$ $= \mathrm{w}_1$	$2w_2 + 2\delta g_2$	$w_2 + 2\delta g_2 + 2ai_2$
θ_T	$\delta + 2f\Omega_1$ $= \delta + \theta_{NT}$	$\delta + 2f\Omega_1$ $= \delta + \theta_{NT}$	$2\delta k + w_2 + 2\delta g_2$ $= 2\delta k + \theta_{NT}$	$2\delta k + w_2 + 2\delta g_2 + 2ai_2$ $= 2\delta k + \theta_{NT}$
V_{Y1}	$2\delta\Omega_1 + \delta w_1$ $+ \mathrm{V}_{F1} + V_\epsilon$	$2\delta\Omega_1 + 2a\Gamma_1 + \delta w_1$ $+ \mathrm{av}_1 + V_{F1} + V_\epsilon$	$2\delta\Omega_1 + \delta w_1 + V_{F1} + V_\epsilon$	$2\delta\Omega_1 + 2a\Gamma_1 + \delta w_1$ $+ \mathrm{av}_1 + V_{F1} + V_\epsilon$
V_{Y2}	$2\delta\Omega_2 + \delta w_2$ $+ \mathrm{V}_{F2} + V_\epsilon$	$2\delta\Omega_2 + 2a\Gamma_2 + \delta w_2$ $+ \mathrm{av}_2 + V_{F2} + V_\epsilon$	$2\delta\Omega_2 + \delta w_2 + V_{F2} + V_\epsilon$	$2\delta\Omega_2 + 2a\Gamma_2 + \delta w_2$ $+ \mathrm{av}_2 + V_{F2} + V_\epsilon$
Γ_1	–	$aj + \frac{1}{2}v_1$	–	$aj + \frac{1}{2}v_1$
Γ_2	–	$aj + \frac{1}{2}v_2$	–	$aj + 2\delta i_2 + \frac{1}{2}v_2 + ah_2$
v_1	–	$2f\Gamma_1$	–	$2\Gamma_1\mu(V_{F1} + \delta w_1 + av_1)$
v_2	–	$2f\Gamma_2$	–	$2f\Gamma_1(1 + V_{Y1}\mu)$
θ_{LNT}	–	$2f\Gamma_1$ $=\mathrm{v}_1$	–	$v_2 + 2\delta i_2 + 2ah_2$
θ_{LT}	–	$2f\Gamma_2$	–	$2aj + v_2 + 2\delta i_2 + 2ah_2$ $= 2\ \mathrm{aj} + \theta_{LNT}$
h_1	–	0	–	0
h_2	–	0	–	$\Gamma_1^2\mu$
i_1	–	0	–	0
i_2	–	0	–	$\Omega\mu\Gamma$ $= \sqrt{g_2h_2}$

Supp. Table II: Disequilibrium Model Constraints (Continued)

	**No AM, No Latent PGS's**	**No AM, Latent PGS's**	**AM, No Latent PGS's**	**AM, Latent PGS's**
cov(Y_p, $L[N]T_m$)<br>cov(Y_m, $L[N]T_p$)	–	0	–	$V_{Y1}\mu\Gamma_1$
cov(Y_p, $[N]T_m$)<br>cov(Y_m, $[N]T_p$)	0	0	$V_{Y1}\mu\Omega_1$	$V_{Y1}\mu\Omega_1$
cov(Y_p, F_m)<br>cov(Y_m, F_p)	0	0	$V_{Y1}\mu(\delta w_1 + V_{F1})$	$V_{Y1}\mu(\delta w_1 + av_1 + V_{F1})$
cov(F_p, F_m)	0	0	$\mu(\delta w_1 + V_{F1})^2$	$\mu(\delta w_1 + av_1 + V_{F1})^2$
cov(Y_p, Y_m)	0	0	$V_{Y1}^2\mu$	$V_{Y1}^2\mu$
cov(Y_o, Y_*)	$\delta\Omega_1 + fV_{Y1}$	$\delta\Omega_1 + a\Gamma_1 + fV_Y$	$(\delta\Omega_1 + fV_{Y1})(1 + V_Y\mu)$	$(\delta\Omega_1 + a\Gamma_1 + fV_{Y1})(1 + V_Y\mu)$
cov(Y_*, F_o)	fV_{Y1}	fV_{Y1}	$fV_{Y1}(1 + V_{Y1}\mu)$	$fV_{Y1}(1 + V_{Y1}\mu)$
cov(Y_*, F_*)	$\delta w_1 + V_{F1}$	$\delta w_1 + av_1 + V_{F1}$	$\delta w_1 + V_{F1}$	$\delta w_1 + av_1 + V_{F1}$

Parameter constraints under disequilibrium. As in Supp. Table I, we use the $*$ subscript to denote either p or m but not both within a single term. We also use subscripts 1 and 2 to denote parent and offspring generations, respectively. For example, g_1 represents the covariance within each parental haplotype (the "cis" correlation; e.g., $cov(T_p, NT_p)$), while g_2 represents the covariance between parental haplotypes (the "trans" correlation; e.g., $cov(T_p, T_m)$).
